# Combined Kalman Filter and Multifeature Fusion Siamese Network for Real-Time Visual Tracking

**DOI:** 10.3390/s19092201

**Published:** 2019-05-13

**Authors:** Lijun Zhou, Jianlin Zhang

**Affiliations:** 1 Key Laboratory of Optical Engineering, Institute of Optics and Electronics, Chinese Academy of Sciences, No.1, Optoelectronic Avenue, Wenxing Town, Shuangliu District, Chengdu 610200, China; zhoulijun16@mails.ucas.edu.cn; 2 University of Chinese Academy of Sciences, Beijing 100000, China

**Keywords:** object tracking, real time, Siamese tracker, Kalman filter

## Abstract

SiamFC has a simple network structure and can be pretrained offline on a large data set, so it has attracted the attention of many researchers. It has no online learning process at all. Hence, there are no good solutions for some complex tracking scenarios such as occlusion and large target deformation. For this problem, we propose a method using the Kalman filter method and fusion multiresolution features and get multiple response scores. The Kalman filter acquires the target’s trajectory information, which is used to process complex tracking scenes and to change the selection method of the search area. This also enables our tracker to stably track fast moving targets.The introduction of the Kalman filter compensates for the shortcomings that SiamFC can only track offline, and the tracking network has an online learning process. The fusion of multiresolution features to obtain multiple response scores map helps the tracker to obtain robust features that can be adapted to a variety of tracking targets. Our proposed method has reached the state-of-the-art in testing on five data sets and can be run in real time (40 fps), including OTB2013, OTB2015, OTB50, VOT2015 and VOT 2016.

## 1. Introduction

Visual tracking aims to estimate the trajectory of a target in a video sequence. It has wide applications ranging from human motion analysis, human–computer interaction to autonomous driving. The main difficulty of video tracking is how to use very limited training data (usually the bounding box in the first frame) to build a tracker that can adapt to various appearance changes, including scale variation, fast motion, occlusions, deformation, and background clutter. It should maintain both stable and real-time tracking.

In recent years, deep object tracking algorithms have achieved very good results in recent benchmarks [1,2] and challenges [3,4], which benefit from the strong feature representation capabilities of convolutional neural networks. Some of the trackers [5,6,7,8] integrate deep features into traditional tracking methods, thus benefitting at once from both the expressiveness of the convolutional neural network (CNN) features and the injected human knowledge implicit in shallow methods. Others [9,10,11,12,13,14,15] simply use a CNN as a classifier and directly deploy end-to-end training. Most of these methods use offline training to improve tracking performance. Unlike handcrafted features, the representation of CNN features in the semantic embedded learning space contains rich high-level semantic information, and it is very effective at identifying objects of different categories. Traditional correlation filter object tracking algorithms use samples extracted from the current tracking video and use online learning methods to model the appearance of the object. However, the obvious drawback of using data derived only from the current tracking video is that only relatively simple models can be learned. To overcome this limitation, some end-to-end object tracking algorithms based on deep learning have been proposed. This achieved good results, which can be mostly attributed to extensive offline pre-training on large datasets, followed by joint training for a smaller number of stochastic gradient descent (SGD) epochs.

In recent years, due to the good performance and speed of SiamFC [16], there have been many follow-up studies based on SiamFC, for example, refs. [17,18,19,20,21,22]. SiamFC [16] considers tracking to be a similarity learning problem. It uses a fully end-to-end object tracking network to train on large offline data sets for higher discriminating power. The correlation filter tracking method is also a tracking method for online learning, but due to the introduction of cyclic displacement, they cause serious boundary effects and affect the accuracy of the tracking process. Unlike the correlation filter tracking method, SiamFC always uses the first frame target as the template for subsequent frame tracking in the same video tracking process. Therefore, it does not adequately deal with the occlusion situation without the process of online learning. It only performs a simple correlation operation to obtain the position of the current frame target during the tracking process, which makes it very dependent on the cosine window that adds to the response score map to filter the background interference around the target in the tracking process. However, the cosine window will have a negative impact on the target of fast motion. These reasons also make SiamFC unable to achieve excellent performance comparable to online trackers in some challenging scenarios. For these limitations, we propose a method of using Kalman filtering to obtain the target motion information and combine it with the Siamese network for stable tracking. This enables a combination of online learning and offline tracking. The motion information is used to resolve the occlusion of the target and change the selection method of the search area during the tracking process. In addition, we also fused multiresolution features to obtain multiple response scores for robust tracking. This paper combines the Kalman filter method not only to take advantage of the temporal–spatial information and motion information it brings. More importantly, it also compensates for the shortcomings of SiamFC not being able to learn online. This is the key reason our method achieves excellent performance. The method solves the difficult tracking caused by fast motion and deformation by changing the selection method of the search area. It can be seen from the results in Section 4.2.1, our method performs well in difficult scenarios.

In summary, we make the following contributions:

- We propose introducing the Siamese network into the Kalman filtering method to obtain the target trajectory information so that the tracker can also perform robust tracking on target occlusion, deformable, and fast motion scenes.
- We improve the tracking network to combine multiple resolution features to obtain multiple response scores map, and then combine them with certain weights. The tracker fuses multiresolution features to make it work better.
- Our tracker performance achieved state-of-the-art status and can be run in real time on OTB [1,2] and VOT [3,4] dataset.

## 2. Related Work

Due to their superior performance, deep features are widely used in computer vision tasks [23,24,25,26,27,28,29]. Previous CNN trackers are based on discriminant or regression models. Discriminant-based tracking methods are refined by online classification. They include support vector machine classification [30], incremental learning [31], and fully connected neural networks [16]. These discriminant trackers require auxiliary training data as well as offline pre-training. On the other hand, regression-based methods typically combine convolution properties with traditional discrimitive correlation filters (DCF) frameworks. DCF can distinguish between templates for any target and its 2D translation. These methods include hierarchical convolution features [8], adaptive hedging [18], spatial regularization [6], and continuous convolution operations [7].

In recent years, researchers have been paying more and more attention to the end-to-end target tracking algorithm. This kind of method can make the tracking performance of the network more robust by performing a large number of offline pretraining on the network. Some works [31,32,33] follow offline training and online fine-tuning, which is time-consuming for real-time tracking. SiamFC [16] uses the siamese network to build a template-based tracker without the need for online updates to achieve higher tracking speeds. There are also some object-tracking methods combined with correlation filter algorithms [33], and the correlation filter layers in these networks also need to be updated online. However, since the derivation of the correlation filter layer is performed in the Fourier frequency domain, the efficiency of the DCF is preserved.

Siamese instance search for tracking(SINT) [19] developed visual tracking as a validation problem and trained a Siamese architecture to learn online target matching metrics. Learning to track at 100 fps with deep regression network(GOTURN) [34] joins pairs of consecutive frames and learns the object tracking state through regression. Convolutional residual learning for visual tracking(CREST) [35] treats the tracking process as a convolution and applies residual learning to explain the change in appearance. FlowTrack [36] uses rich flow information in successive frames to improve feature representation and tracking accuracy. CFNet [33] interprets the correlation filter learner as a differentiable layer in a deep neural network. SA-Siam [22] consists of a semantic branch and an appearance branch, each of which is a similarity learning dual network for real-time target tracking based on the Siamese network structure. RASNet [37] has added attention mechanisms (including residual attention, general attention, and channel attention) based on the Siamese network structure. This attention mechanism is embedded as a layer in the Siamese network, which alleviates the overfitting problem in deep network training. It also enhances the discriminating ability and adaptability of the network. These methods are designed to enhance the representation of features, but they are still not robust to accurate tracking of difficult targets in some difficult scenarios, such as fast motion, occlusion, and so on. Inspired by these limitations, we decided to solve this problem by combining the Siamese tracking network with the Kalman filter.

The Kalman filter is used in the target tracking field due to its good performance. It keeps track of the target or assists the tracking process by constantly updating the state of the target. Speed and Hardware Optimization of ORDP Algorithm Based Kalman Filter for 2D Object Tracking [38] introduces a Kalman filter for tracking the desired dynamic object and filtering the noise in the 2D object tracking by estimating the state of the object. Video object tracking using an adaptive Kalman filter [39] sets the system model of the adaptive Kalman filter to construct the motion model in the tracking process and uses the dominant color of the moving object in the Hue-Saturation-Intensity(HSI) color space as the feature of detecting the moving object in the continuous video frame. Object tracking using an adaptive Kalman filter combined with mean shift [40] proposes a binding mean shift (MS) of the adaptive Kalman filter (KF) of the target tracking algorithm. The system model of KF is constructed in this paper, and the center of the object predicted by KF is used as the initial value of the MS algorithm. Optimized Neural Network Parameters Using Stochastic Fractal Technique to Compensate Kalman Filter for Power System-Tracking-State Estimation [41] discusses the Kalman filtering enhanced by the random fractal search technique based on optimized neural network parameters. KF gain (mismodeling error) and measurement noise are replaced by an optimized multilayer perceptron (MLP-SFS). Real-time vehicle detection and tracking in video based on faster R-CNN [42]: In this paper, a detection method based on deep learning fast R-CNN is proposed to solve the problem of vehicle detection and tracking in complex scenes.This method combines the Camshift and Kalman filter algorithms for vehicle tracking. These methods use the Kalman filter to directly track the object. When only the Kalman filter is used for tracking, there may be cases where the target tracking is lost. We took advantage of the stability of the deep target tracker results and used the position obtained by the deep tracker as an observation of the Kalman filter to make it more stable. We mainly used the Kalman filter to supplement the tracking network to cope with the occlusion of the target and the selection of the search area instead of tracking the object with the Kalman filter alone.

## 3. Method

The method proposed in this paper is mainly inspired by the problem of not being able to stably track fast moving and occluded targets in SiamFC. We do not want to introduce an online learning method for correlation filtering to solve this problem, because the correlation filtering will bring the boundary effect to limit the performance of the tracker. Our approach is to introduce a Kalman filtering method to make a stable prediction of the target’s motion trajectory, and the target search area in the tracking process is tailored according to the target position obtained by Kalman filtering, instead of cropping the search area with the position of the previous frame target as in the previous method. This cropping method can avoid the case where the fast moving target is cropped in the search area or the target is filtered by the cosine window. At the same time, we also used the acquired motion information to guide the tracking process to cope with the situation where the target is tracked. When we detect that the target is severely occluded, we no longer use the network to track the location to update the current position but use our motion prediction. The location to which the current target is updated. This solves the problem of the target being lost when the target is occluded.

Finally, in order to make full use of multiresolution features, we applied different resolution features of different layers to predict the response scores map of the targets and combined them with certain weights.

### 3.1. Network Architecture

**Figure 1:**
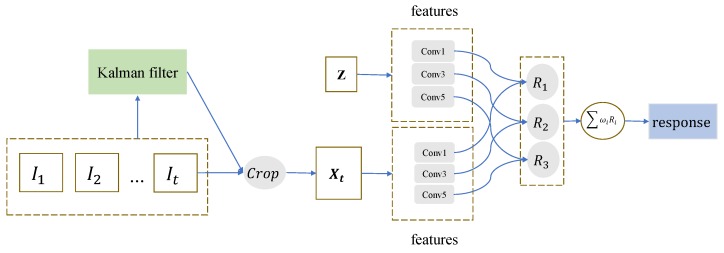
The architecture of the proposed Kalman–Siam network. It consists of a Kalman filter motion trajectory estimation module and a tracking network. The input of the motion trajectory prediction module is an image of the current frame and the previous frame, which predicts the position of the target at the current frame and cropping the search area. In the tracking network module, the templates and the different resolution features of the search image are respectively subjected to correlation operations to obtain response score maps of different layer features. The response score maps are finally combined with a certain weight.

We propose the framework as shown in Figure 1. The Kalman filter module inputs are $I_{1}$, $I_{2}$, …, $I_{t}$. They represent 1 to *t* frame video images, where the *t*th frame represents the current frame image. It is used to predict the trajectory information of the target to revise the tracking results of the tracking network and the cropping of the search area to cope with the fast motion and occluded targets in the tracking process. The tracking network input is a pair which is a tracking template cropped from the first frame and a search area cropped from the current *t*th frame image. They are called *z* and $X_{t}$. The sizes of *z* and $X_{t}$ are 127 × 127 and 255 × 255, respectively.

First, our network uses a Kalman filter to predict the trajectory of the target to determine the motion information of the target in the current frame. The target position obtained by the tracking network is the observation value of the Kalman filter module. We selected the joint features of the first, third, and fifth layers in the feature extraction network to predict the tracking results. The purpose of this is to make full use of the multiresolution feature information to achieve stable target tracking. The features of multiresolution features are different, and combining this multilevel feature information will greatly benefit the tracking process. The feature information from the shallow to the deep of the CNN layer has specific performance for the tracking problem. The shallow features have rich details, better adaptability and precision, and good performance for precise positioning of targets. Deeper features have rich semantic features for better stability when dealing with larger deformations.

The fully-convolutional Siamese network uses a pre-offline learning approach to train a neural network to solve the problem of general similarity learning. Image *X* represents the search image, typically the current video frame, and *z* represents the object’s template. *X* and *z* pass through the two branched CNN networks and then cross-correlate in the feature space. This process can be expressed as the following equation:

(1) R(z,X)=Corr(ϕ(z),ϕ(X)),

where ϕ(·) is the feature representation. corr(·) is the correlation operation. R(z,X) denotes the similarity between the target image and the search image. It is a response score map generated by the template and search area correlation operations. Kalman–Siam is optimized by minimizing the logistic loss during the training process.

(2) $$arg\min_{\theta }\frac{1}{\left|N\right|}\sum_{i=1}^{N}Loss\left(y_{i},R\left(z_{i},x_{i};\theta \right)\right),$$

where θ is the parameters in SiamFC, N is the number of training samples. R(·) is the maximum response score to the pair $\left(z_{i},x_{i}\right)$. $y_{i}$ is the label of the search image indicating whether it is a positive sample or a negative sample. D is the number of training samples. Function Loss(·) is as follows:

(3) $$Loss=\frac{1}{\left|D\right|}\sum_{v\in D}\log\left(1+\exp\left(-y\left(v\right)\cdot R\left(v\right)\right)\right),$$

where R(v) is score map for each position v∈D. y(v)∈{+1,−1} is ground truth label for each position v∈D. Our network uses multiresolution features to calculate the response scores of the tracking separately, and the response scores are obtained by summing them with some weights. It fully utilizes the functions of different resolution features for stable tracking. This response score is as follows:

(4) $$R=\sum_{i}\left(\omega_{i}\cdot R_{i}\right),$$

where *i* is the number of multiple feature. We set it to 3-layer. $\omega_{i}$ is the weight of each layer feature. We set them to 0.2, 0.2, and 0.6, respectively.

### 3.2. Temporal–Spatial Features with Kalman Filter

As all we know that the object tracking is a process involving both time dimension and space dimension, obviously, it would benefit from temporal–spatial features. However, the original SiamFC just focuses on the spatial feature, whose correlation filtering rolls over the feature map will be undermined by the background clutter. Therefore, for the purpose of representing the object with a temporal–spatial feature, we propose this new method Kalman–Siam by combining the Kalman filter with SiamFC, which integrates object temporal trajectory information with object deep spatial features from the Kalman filter and deep network, respectively. By adopting the Kalman filter, Kalman–Siam can directly choose the candidate object area for SiamFC’s correlation operation and can naturally cope with fast moving object tracking. It is somewhat similar to the method in [43], which employs the particle filter to the deep network. However, Kalman–Siam is different from that method for two reasons, the first being that Kalman filter is more efficient than the particle filter, especially for real-time tracking, and the second being that the deep network can help to discriminate the object from the background, which mitigates the dependence of prediction.

We used the Kalman filter to capture the trajectory information of the target between the video frames to make full use of the time information. This trajectory information can process the target occlusion situation in the tracking and accurately crop the search area. The Kalman filter is a recursive process. It mainly has two update processes, including time update and observation update. The time update is mainly for the prediction of the system, including state prediction and covariance prediction, so it is also called a prediction module. The observation update includes calculating the Kalman gain, status update, and covariance update, also known as the correction phase. The Kalman filter uses the observations of the current state to correct the predicted values obtained during the prediction phase to obtain a new estimate that is more closely connected to the real value. Therefore, the entire recursive process mainly includes five aspects of calculation. First, it is a prediction of the state:

(5) $$\hat{x}\left(k|k-1\right)=\phi_{k}x\left(k-1|k-1\right)+B_{k}U\left(k\right),$$

where $\hat{x}\left(k|k-1\right)$ is the process state at time *k*, U(k) is the control amount of the process at time *k* (if there is no control, it can be zero), ϕ is the state transition matrix, and *B* is the system parameter. x(k−1|k−1) is the optimal result of the previous state, and $\hat{x}\left(k|k-1\right)$ is the result obtained by the previous state prediction. In order to conveniently use the state transition matrix, the state *x* of the Kalman filter is to include the coordinates *x*, *y*, and the velocity $v_{x}$, $v_{y}$ of the target. The main purpose of this step is to update the process results. Then, it is to predict the covariance corresponding to the process result:

(6) $$\hat{p}\left(k|k-1\right)=\phi_{k}p\left(k-1|k-1\right)\phi_{k}^{T}+Q,$$

where p(k−1|k−1) is the covariance corresponding to the previous state x(k−1|k−1), and $\hat{p}\left(k|k-1\right)$ is a prediction of the covariance corresponding to the state x(k|k−1), *Q* is the covariance matrix of system process noise (assumed to be Gaussian white noise).

Next is the observation update. First, we need to calculate the Kalman gain for status updates and covariance updates. Since the first two predictions get the prediction result of the current state, and then the observation value of the current state is obtained, the state state update can be realized by obtaining the optimal state estimation of the current state through the prediction value, the observation value, and the gain.

(7) $$g_{k}=\frac{p\left(k|k-1\right)H^{T}}{Hp\left(k|k-1\right)H^{T}+R}\;,$$

where $g_{k}$ is the Kalman gain, *H* is the observation matrix, and *R* is the covariance matrix corresponding to the measurement noise (also assumed to be Gaussian white noise).We have obtained observations and predicted values and gains for the current state. The state update uses them to calculate the optimal state estimate x(k|k) at time *k*.

(8) $$x\left(k|k\right)=x\left(k|k-1\right)+g_{k}\left(Z\left(k\right)-Hx\left(k|k-1\right)\right),$$

where Z(k) is the observed value at time *k*. Finally, the covariance update is performed, and the state has been updated. In order to ensure that the process loops. Expressed as:

(9) $$p\left(k|k\right)=\left(I-g_{k}H\right)p\left(k|k-1\right)),$$

where *I* is a unit matrix. We have got the trajectory information of the target and use it to guide our tracking process. It changes the way the search area is selected during tracking. Instead of cropping using the center of the target in the previous frame, it uses the target position predicted by the Kalman filter for cropping. If there is an occlusion in the tracking process, this trajectory information is used as the tracked location, not the location of the network output, because a tracking network without an online learning process cannot deal with occlusion problems. We introduce how to determine if the target is occluded in the next section.

### 3.3. Occlusion Update

The Siamese network is a completely offline method of tracking of the process without online learning. The template for the similarity search of the search area is always the target in the first frame. The advantage is that we do not have to worry about the template being polluted, since the template is always the ground truth given in the first frame. However, when the target encounters large deformation or occlusion, the Siamese network cannot track these situations stably.

In order to solve these situations, we propose using the Kalman filter to predict the trajectory information of the target to compensate for the defects of the network. When we find that the target is occluded by the occlusion discrimination method, the target position predicted by the Kalman filtering method is updated for the target position of the current frame. This can avoid tracking the network to inaccurate target tracking or tracking failure. This is to deal with complex tracking scenes where the target is occluded or deformed. The occlusion discrimination method utilizes the peak signal-to-noise ratio of the response score map. We normalized the value of the response score map to 0–1 and set a threshold if the maximum response score of the current frame image is less than this threshold. We then used the occlusion discrimination method to determine the difference between the image response scores of the previous frames and the following equations:

(10) $$P=\frac{R_{peak}-R_{low}}{mean(R\setminus R_{peak})},$$

(11) $$P_{ave}=\frac{P(t-1)+P(t-2)+P(t-3)+P(t-4)}{4},$$

where $R_{peak}$ is the maximum value in the *t*th frame response score map, and $R_{low}$ is the minimum value in the *t*th frame response score map. When $R_{peak}$ and the ratio of $P/P_{ave}$ are simultaneously less than the respectively set threshold. Our update to the target location does not use the target location tracked by the network. This location may be affected by factors such as occlusion or deformation, and the tracking may be inaccurate. Thus, we updated the position tracked by the Kalman filter as the current frame target position.

## 4. Experiments

### 4.1. Implementation Details

Our approach is to use only color images offline training on the ILSVRC-2015 [44] video dataset. ILSVRC-2015 [44] has more than 4000 sequences in total, with approximately 1.3 million frames and approximately 2 million tracking objects with ground truth bounding boxes. We randomly selected a pair of images from a video and crop *z*, where *z* is at the center and *X* is cropped from another image, where the ground truth of the target is centered. The gradient was adjusted using the SGD. These parameters were originally initialized by a modified version of Xavier, with a small batch size of 8. The network trained for 50 epochs, and the learning rate dropped from 0.01 to 0.0001 in logarithmic form.

In the inference phase, the three network branches are combined by weights. Through our experiments, the tracker can get the best performance when the weights are 0.2, 0.2, and 0.6. High-level features with rich semantic information can adapt to some of the larger transformations of the target, having obvious advantages for the recognition of the target. Thus, we have higher weights for high-level features. Low-level features with high-resolution information have clear advantages for target recognition. The low-level features are mainly to accurately locate the position of the target after the high-level features have identified the target. In order to prove the effectiveness of our method, rather than the results obtained by fine-tuning the parameters, for the selection of different layer response score fusion parameters, we conducted a simple experimental test without a too-fine parameter search. We always kept the weight of the feature of the 5th layer greater than 0.5, and the result of the selected parameter test fluctuated within 0.01. This was evaluated on several selected videos on the visual tracker benchmark(OTB).In the occlusion discrimination, the threshold of the response score and the ratio of $P/P_{ave}$ are 0.3 and 0.6, respectively. When it is less than this threshold, we determine that the target is suddenly occluded or encounters a large deformation. The proportional change step between different scale searches is 1.0375, the damping factor for scale update is 0.59, and the score penalty for scale change is 0.9745, and the weight of the cosine window for penalizing large displacement is 0.176. The search is performed on three scales during tracking. We implemented our model in the TensorFlow 1.2.1 framework. Our experiments were performed on a PC equipped with a Xeon E5 2.4 GHz CPU and a GeForce GTX 1080 Ti GPU. The average test speed of the method is 40 fps.

### 4.2. Comparison with State-of-the-Arts

We compared Kalman–Siam with the state-of-the-art real-time trackers on five benchmarks, including OTB-50, OTB-2013, OTB- 2015, VOT2015, and VOT2016.

#### 4.2.1. Results on OTB

The OTB2013 dataset [1] is one of the most widely used normative datasets in visual tracking, and it contains 51 sequences of images with various challenging factors. Its evaluation is based on two indicators: The precision plots and the success plots. The average overlap precision (OP) is defined as the percentage of frames in the video where the intersection exceeds a threshold of 0.5. The area under the curve (AUC) of each success plots is used to rank the tracking algorithm. The AUC is calculated from the success plots where the average OP of all videos is plotted within the threshold [0, 1]. The OTB2015 [2] dataset is an extension of the OTB2013 dataset, which contains 100 video sequences. The OTB 50 is a relatively difficult video sequence in OTB 100.

**Figure 2:**
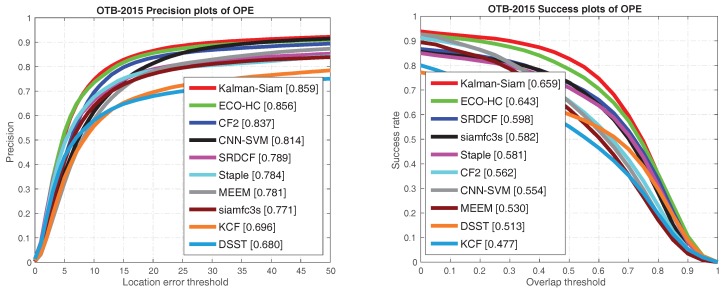
The precision plots and success plots on OTB-2015 benchmarks. The curves and numbers were generated with an visual tracker benchmark(OTB) toolkit.

**Figure 3:**
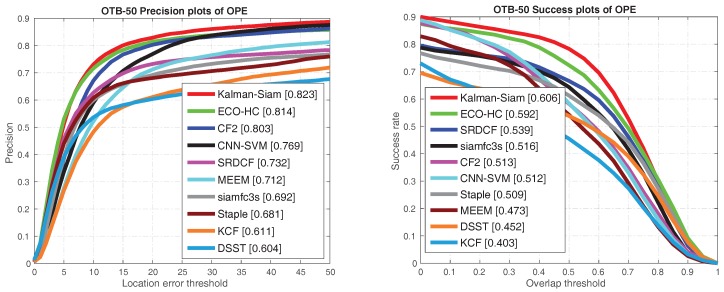
The precision plots and success plots on OTB-50 benchmarks. The curves and numbers were generated with an visual tracker benchmark(OTB) toolkit.

**Figure 4:**
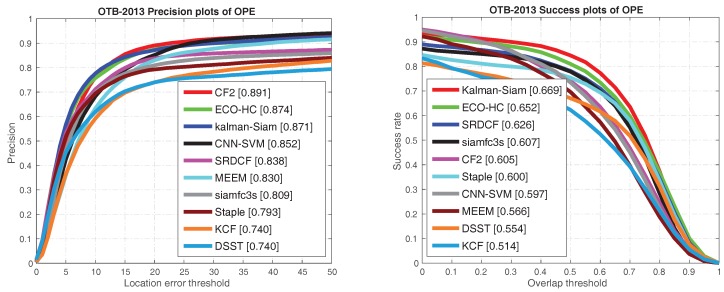
The precision plots and success plots on OTB-2013 benchmarks. Curves and numbers are generated with visual tracker benchmark(OTB) toolkit.

**Figure 5:**
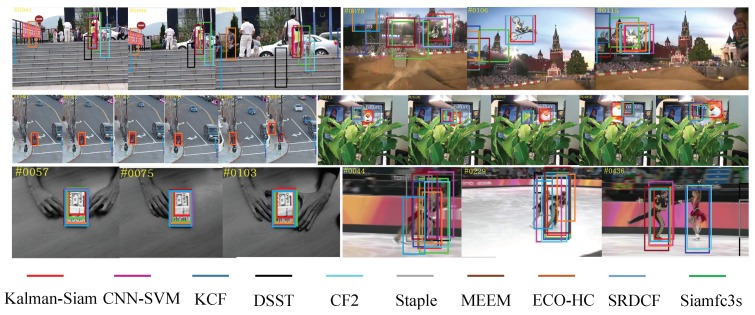
Qualitative results of our Kalman–Siam, along with state-of-the-art trackers on six challenge sequences. *girl2, motorRolling, Human3, tiger1, coupon, skating2*. Kalman–Siam tracks accurately and robustly in these hard cases.

**Figure 6:**
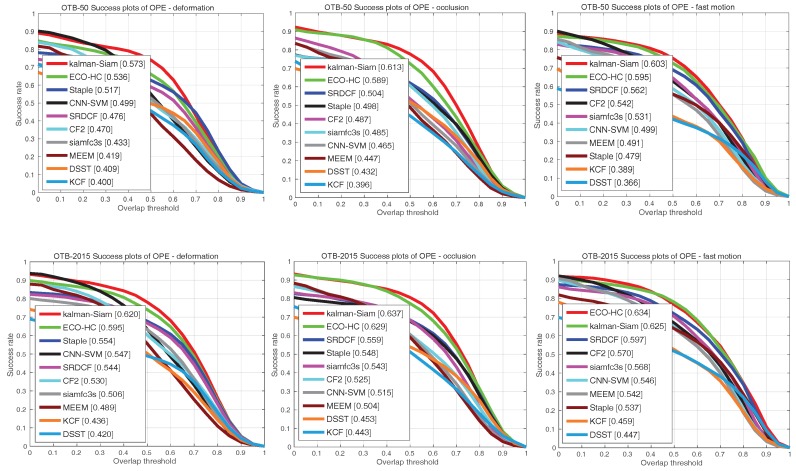
The success plots on the OTB-2015 and OTB-50 dataset in the three scenarios of occlusion, fast motion, and deformation.

The Kalman–Siam tracker is compared with recent state-of-the-art trackers, including SiamFC3s [16], end-to-end representation learning for correlation filter based tracking(CF2) [33], efficient convolution operators for tracking(ECO-hc) [5], complementary learners for real-time tracking(Staple) [45], online tracking by learning discriminative saliency map with convolutional neural network(CNN-SVM) [30], robust tracking via multiple experts using entropy minimization (MEEM) [46], convolutional features for correlation filter based visual tracking (SRDCF) [6], high-speed tracking with kernelized correlation filters(KCF) [47], and accurate scale estimation for robust visual tracking(DSST) [48] on OTB 2015, OTB50, and OTB2013 benchmarks. The precision plots and success plots of one path evaluation (OPE) are shown in Figure 2, Figure 3 and Figure 4. The comparison shows that Kalman–Siam achieves the best performance among these real-time trackers on all three OTB benchmarks. The Kalman–Siam tracker achieves an AUC score of 65.9%, 60.6%, and 66.9% on OTB2015, OTB50, and OTB2013, respectively, at real-time speed (40 fps). Compared to the SiamFC trackers [16], our tracker obtains significant AUC gains of 7.9%, 9.0%, and 6.2% on OTB2015, OTB50, and OTB2013, respectively. The results prove that the method of Kalman filtering and a multifeature fusion siamese network can be stable and accurate. Figure 5 shows some qualitative results of our tracker compared with 9 real-time trackers. We compared under the curve (AUC) scores and speeds in the OTB benchmark with the state-of-the-art trackers, shown in Table 1. Kalman–Siam also has the speed of a real-time operation while maintaining the highest accuracy. In order to prove that our method has good performance in dealing with three difficult scenes of target occlusion, fast motion and deformation. we show here the OTB-2015 and OTB-50 success plot in the three scenarios of occlusion, fast motion, and target deformation. It can be seen from Figure 6 that our method, Kalman–Siam, has always maintained good performance in these difficult scenarios.

#### 4.2.2. Results on VOT

In the visual object tracking (VOT) benchmark, we chose VOT2015 and VOT2016 to evaluate our algorithm. VOT2015 [3] contains 60 challenging videos. In VOT2016 [4], 10 sequences from VOT2015 are replaced by new sequences. The visual object tracking (VOT) challenge is a competition between short-term model-free visual tracking algorithms containing 60 sequences. For each sequence in the data set, the tracker is evaluated by the rectangular initialization of the target in the first frame. As long as the target is lost, the toolkit will restart the tracker. Robustness is obtained by calculating the average number of failures, and the accuracy is the statistical average crossover ratio.

**Figure 7:**
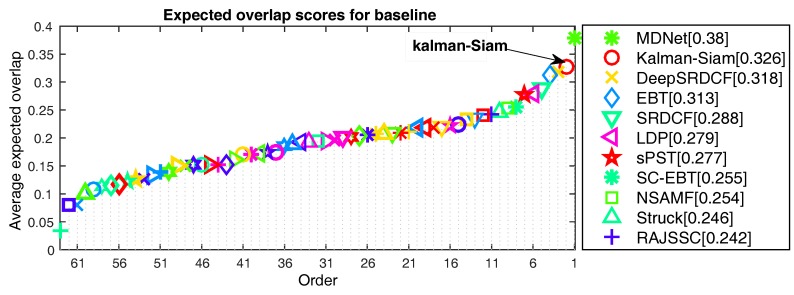
Expected average overlap(EAO) ranking with trackers in VOT2015. The legend shows the results of the top 10 tracker and Kalman–Siam. The yellow horizontal dotted line indicates the VOT2015 state-of-the-art bound.

**Figure 8:**
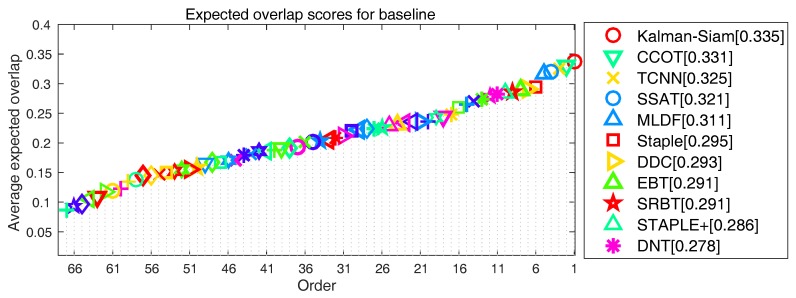
Expected average overlap(EAO) ranking with trackers in VOT2016. The legend shows the results of the top 10 tracker and Kalman–Siam.

In the VOT2015 [3] and VOT2016 [4] experiments, we present a state-of-the-art comparison with the participants in the challenge. The expected average overlap(EAO) curve evaluated at VOT2015 is shown in Figure 7, comparing 62 state-of-the-art trackers. The results of Kalman–Siam are comparable to those of the most advanced algorithms, and the resulting EAO score of 0.326 is the second best result. MDNet [11] is not compatible with the latest VOT rules because of OTB training data. Our tracker does not use any tracking benchmarks in offline training. The EAO curve evaluated at VOT2016 is shown in Figure 8. it illustrates that our tracker ranks 1st among 70 trackers according to the EAO standard. The resulting EAO score of 0.335 is the best result. In addition, our algorithm can run in real time (40 fps).

### 4.3. Ablation Analysis

In this section, we described how we performed an ablation analysis to demonstrate the effectiveness of the proposed component. We compared the AUC scores on the three datasets, OTB2015, OTB2013, and OTB50. We used SiamFC as a baseline and then added the proposed components. There are several variants of the algorithm. Multi-Siam is a method of adding multiresolution multiresponse score fusion to the baseline. Kalman–Nomuti–Siam is a method in which the Kalman filter is used at the baseline to deal with occlusion and large deformation, and the crop method of the search area is changed. Kalman–Siam represents the final algorithm of the proposed algorithm with a Kalman filter and multiresolution feature fusion. From the results in Table 2, it can be seen that the gain of the AUC score of multi-Siam was compared with the gain of the AUC score of a SiamFC of 2.6%, 4.0%, and 2.5%. Kalman–Nomuti–Siam was compared with a SiamFC of 1.5%, 2.9%, 2.4%. The gain of the AUC score of Kalman–Siam was compared with a SiamFC of 7.9%, 9.0% and 6.2%. The performance of our method has increased dramatically. This experiment proves that the components we added are very efficient and have good performance in solving problems in the tracking network.

## 5. Conclusions

In this paper, we proposed a combined Kalman filter and multifeature fusion Siamese network for a real-time object tracking network. In the framework, we used the Kalman filter to predict the trajectory of the tracking target, and tracked the position of the tracking of the network as an observation to revise the Kalman filter. Our proposed method enables the tracking network to robustly deal with complex tracking scenes such as fast motion, target occlusion, deformation, and more. The network also combines multiple resolutions features to generate multiple response score maps for stable tracking. The tracker was tested on five public datasets, and our method achieved outstanding gain relative to the baseline and reached state-of-the-art status.

## Figures and Tables

**Table 1:** Comparison area under the curve (AUC) scores and speeds in the OTB benchmark with the state-of-the-art trackers.

	Tracker	Ours	ECO-HC	SRDCF	SiamFC	CF2	Staple	MEEM	DSST	KCF
OTB-2013	AUC	0.669	0.652	0.626	0.607	0.605	0.600	0.566	0.554	0.514
OTB-50	AUC	0.606	0.592	0.539	0.516	0.513	0.509	0.473	0.452	0.403
OTB-2015	AUC	0.659	0.643	0.598	0.582	0.562	0.581	0.530	0.513	0.477
	FPS	40	60	5	86	75	80	10	24	172

**Table 2:** Ablation study of the effectiveness of tracking components on OTB using the area under the curve (AUC).

Tracker	OTB2013	OTB2015	OTB50
SiamFC	60.7	58.2	51.6
Multi-Siam	63.33	62.21	54.13
Kalman–Nomuti–Siam	62.26	61.10	54.01
Kalman–Siam	66.9	65.9	60.6
